# Facilitating and limiting factors of cultural norms influencing use of maternal health services in primary health care facilities in Kogi State, Nigeria; a focused ethnographic research on Igala women

**DOI:** 10.1186/s12884-024-06747-x

**Published:** 2024-08-27

**Authors:** Uchechi Clara Opara, Peace Njideka Iheanacho, Hua Li, Pammla Petrucka

**Affiliations:** 1 College of Nursing, University of Saskatchewan, Health Science Building - 1A10, Box 6, 107 Wiggins Road, Saskatoon, Saskatchewan, SK S7N 5E5​ Canada; 2 Department of Nursing Sciences, University of Nigeria, Enugu Campus, Enugu, Enugu State Nigeria

**Keywords:** Facilitating, Limiting, PEN 3 cultural model, Cultural norms, Primary health facilities, Nigeria

## Abstract

**Background:**

Facilitating factors are potential factors that encourage the uptake of maternal health services, while limiting factors are those potential factors that limit women’s access to maternal health services. Though cultural norms or values are significant factors that influence health-seeking behaviour, there is a limited exploration of the facilitating and limiting factors of these cultural norms and values on the use of maternal health services in primary health care facilities.

**Aim:**

To understand the facilitating and limiting factors of cultural values and norms that influence the use of maternal health services in primary healthcare facilities.

**Methods:**

The study was conducted in two primary healthcare facilities (rural and urban) using a focused ethnographic methodology described by Roper and Shapira. The study comprised 189 hours of observation of nine women from the third trimester to deliveries. Using purposive and snowballing techniques, data was collected through 21 in-depth interviews, two focus group discussions comprising 13 women, and field notes. All data was analyzed using the steps described by Roper and Shapira (Ethnography in nursing research, 2000).

**Results:**

Using the enabler and nurturer constructs of the relationships and the expectations domain of the PEN-3 cultural model, four themes were generated: 1, The attitude of healthcare workers and 2, Factors within primary healthcare facilities, which revealed both facilitating and limiting factors. The remaining themes, 3, The High cost of services, and 4, Contextual issues within communities revealed factors that limit access to facility care.

**Conclusion:**

Several facilitating and limiting factors of cultural norms and values significantly influence women’s health-seeking behaviours and use of primary health facilities. Further studies are needed on approaches to harness these factors in providing holistic care tailored to communities' cultural needs. Additionally, reinvigoration and strengthening of primary health facilities in Nigeria is critical to promoting comprehensive care that could reduce maternal mortality and enhance maternal health outcomes.

**Supplementary Information:**

The online version contains supplementary material available at 10.1186/s12884-024-06747-x.

## Background

Access to maternal health services (MHS) during pregnancy, labour and puerperium is a significant global mandate that has informed the development of maternal health strategies globally. Maternal health strategies are crucial as more than 80% of all pregnancy-related maternal mortality is preventable if adequate, timely and evidence-based MHS is made available to women around childbirth [1]. Consequently, to enhance maternal health outcomes, the Sustainable Development Goals (SDG) were developed, with goal #3:1 focusing on strengthening the health system in reducing maternal mortality, with two indicators aiming to reduce maternal mortality ratio (MMR) and increase skilled birth attendants (SBAs) at birth [2]. Specifically, SDG# 3:1 aims to reduce the maternal mortality ratio to less than 70 per 100,000 live births by 2030 [3]. While approximately 83% of women access SBAs at birth globally, in Sub-Saharan Africa (SSA), only 70% of women access SBAs at birth, and these rates could be even lower in some SSA countries such as Nigeria (United Nations International Children’s Emergency Fund [4].

Nigeria has one of the highest maternal mortality ratios of 1047 per 100,000 live births, with approximately 61% of women having access to SBAs in urban areas and only 26% in rural areas in 2018 [5], despite maternal health policies such as the Integrated Maternal Newborn and Child Health Strategies (IMNCH) and the Midwives Service Scheme (MSS) developed to enhance access to MHS [6–8]. However, the World Health Organization emphasized that the current global evidence of MMR status is calculated based on the estimate from the COVID-19 pandemic [1], which contributed significantly to increased maternal mortality due to limited access to maternal healthcare provision globally as well as in Nigeria [1, 9–12]. Thus, the Nigerian MMR is unclear due to a lack of current national evidence on maternal mortality.

In Nigeria, delays in seeking facility care, reaching a health facility, and receiving expert care were seen as major contributing factors to maternal mortality [13–15]. Systemic factors, such as lack of access to facility care, lack of awareness of the importance of MHS, limited health infrastructure, and cultural factors, are significant factors that limit women’s access to facility care [13, 16, 17].

Several studies have identified cultural norms and practices associated with the use of traditional medicine, reliance on intergenerational values around pregnancy and birth, patriarchal norms and home deliveries as significant factors that limit access to MHS in Nigeria [16–21]. These cultural norms and values are intergenerational standards, beliefs, and practices characterized by distinct cultural features in religion, health, illness and language, which could influence people’s health-seeking behaviour [18, 20–22]. For example, a cultural group’s interpretations of health and illness could contradict Western medical opinions or interpretations and may not always be shared within Western health facilities [23, 24]. Thus, maternal health issues may not easily be diagnosed, which could delay the detection of preventable maternal health issues in Western facilities while favouring traditional and alternative healers, given the cultural interpretation of such health issues [23–26]. While some cultural beliefs and practices are beneficial and should be encouraged, others could be harmful and negatively influence women’s use of MHS and maternal health outcomes [25, 27, 28].

Studies also reveal that the safe motherhood initiative developed by the Nigerian government to increase access to facility care and reduce maternal mortality was hindered by several factors, including cultural beliefs and values that limited women’s access to quality MHS, especially in rural areas, resulting in significant maternal mortality [13, 19, 29]. Regardless, there is a limited understanding of the facilitating and limiting factors of these cultural beliefs and practices. Facilitating factors are potential factors that encourage the uptake of MHS, while limiting factors are those potential factors that limit women’s access to MHS.

Limited use of MHS is found to be significant in primary health care facilities (PHC), which are the first tier of the three-tiered health systems in Nigeria. These PHCs are located within five kilometres of residential areas and are considered the first point of call for all Nigerian citizens in rural and urban areas [18, 30–32]. The PHCs are designed to provide comprehensive MHS ranging from preventive, curative, and promotive services that are timely and safe, without the barriers of cost, culture, and geographical limitations [19, 30, 31]. A functional PHC facility provides a conducive environment that ensures adequate and comprehensive provision of MHS through the availability of human and material resources in the form of health personnel, medical supplies, and adequate health financing [17, 18, 32, 33]. A wide range of people, such as stakeholders, policymakers, healthcare providers, leaders, and representatives of health institutions, as well as community and traditional leaders, make decisions that inform the effective functioning of PHC facilities [19, 34]. According to [35], only 20% of the total 30,000 PHCs in Nigeria are functional, limiting access to quality MHS to a vast population of women. Such limitations could account for women’s preference for traditional practices and traditional birth attendants who live within communities and are believed to provide culturally appropriate care to women around childbirth [18, 21].

Though studies have emphasized the significance of culture in influencing quality access to maternal health [17–19], there is a limited understanding of the facilitating or limiting factors of cultural norms and values that influence the use of MHS in PHC facilities. Consequently, based on these imperatives, understanding women’s perspectives and experiences of these facilitating and limiting factors of cultural norms or values that influence women’s use of MHS in PHC is crucial as such could be harnessed to inform culture-centred maternal health strategies and interventions that could enhance women’s use of MHS and achievement of the SDG #3:1 in 2030. To promote an in-depth understanding of these facilitating and limiting factors, the PEN-3 cultural model was used as a framework to organize our findings, which provided a deep exploration of the facilitating and limiting factors of cultural norms and values that influence women’s use of MHS in PHCs. The PEN-3 cultural model is a culturally-focused model that has been used to explore and understand cultural issues related to health and illness in many countries, including Nigeria [36, 37], where the study was conducted.

## The PEN-3 cultural model

The PEN-3 cultural model is made up of three domains, namely (1) cultural identity (person, extended family, neighbourhood), (2) relationships and expectations (perceptions, enablers, and nurturers) and (3) cultural empowerment (positive, existential, and negative [38, 39]. The cultural identity domain is the port of entry for health interventions, which could be at the level of persons (mothers or health care workers), extended family members (grandmothers), or neighbourhoods (communities or villages) [40]. In the relationships and expectations domain, which forms a part of the assessment phase, people’s perceptions or attitudes concerning health issues, social structures or facilities, such as health services that enhance or limit effective health-seeking practices, and the part played by the family and kin in health-seeking decision related to use of health facilities is explored [39, 40]. The cultural empowerment domain forms the second assessment domain, where health issues are explored to understand and highlight those that are positive [40]. The existential factors or factors without harmful effects are explored before the negative health beliefs and practices are explored and discouraged during the intervention phase [39]. The enabler and nurturer constructs of the relationships and expectations domain guided this study.

### Aim

The study aimed to understand the facilitating and limiting factors of cultural values, norms, and practices influencing the use of MHS in PHC facilities among the Igala ethnicity in Kogi state, Nigeria.

### Design

The focused ethnographic methodology described by nurse anthropologists Roper and Shapira [41] guided this study. Focused ethnography focuses on exploration of a distinct phenomenon within a subculture using narrow research questions. One aim of focused ethnography is to explore health beliefs, values, and practices within a specific subculture of a population in which such experiences occur [41]. Findings from focused ethnographic research could be integrated into nursing practice to enhance health outcomes [41].

The focused ethnography described by Roper and Shapira [41] was deemed appropriate for the study because, unlike other qualitative methodologies, the philosophical underpinning of focused ethnography is grounded on culture and on understanding the subculture of a population [41], which is the focus of this study. In addition, while many qualitative approaches may have some similarities in ontological, epistemological and methodological approaches, the aim, research questions and methods employed in answering such questions differ [42]. Thus, using Roper and Shapira’s [41] focused ethnography, which allows for diverse methods in data collection, enhanced a rich exploration of our research question, leading to a deep understanding of the facilitating and limiting factors of cultural norms and values that influence the use of MHS in PHC facilities in Kogi state Nigeria.

Using Roper and Shapira’s [41] focus ethnographic methodology allowed for engagement in short-term and targeted data-gathering sections with participant observations, individual interviews, field notes, and focused group discussions in the fieldwork, specifically structured to fit the research question. The authors strove for rigour by using diverse data triangulation approaches such as interviews, focused group discussions, participant observations, field notes, reflective memo, and analysis of all documents gathered in the research context. This approach provided us with a thick understanding and interpretation of the facilitating and limiting factors of cultural norms and values influencing the use of MHS in PHCs. This study was reported according to the Standard for Reporting Qualitative Research (SRQR) [43]. In addition, this study is part of a larger study conducted to understand the cultural beliefs and practices of Igala women influencing their use of MHS in PHC facilities in Kogi state Nigeria.

### Positionality

The first and second authors are Nigerian clinical nurse midwives and researchers who have worked among the Igalas in Kogi state, Nigeria, for the past 25 years. Both authors understand the Indigenous Igala language and speak pidgin English (an English-based Indigenous language spoken as a lingua franca in Nigeria). The first author practiced as a clinical nurse midwife in a secondary health care facility among the Igalas in Kogi state for 25 years and has carried out several qualitative studies in Igala land. The second author, who has extensive qualitative research experience, is a professor at a university in Nigeria. The third author is a Canadian professor with years of experience in maternal health research in middle and low-income countries. Finally, the anchor author is a Canadian professor with immense experience in maternal health research in Africa and other low- and middle-income countries and is the overall supervisor of the research. The first author (who collected the data) ensured reflexivity, given her exposure to the ethnic group and her role in data gathering, which could influence the research and data analysis processes.

### Reflexivity

The researchers put in place a range of approaches to enhance reflexivity. The first author’s position as an insider allowed her to assume the diffractive way of knowing, which positioned her as an active participant in knowledge creation and not one she distanced from, knowing that knowledge creation is an entangled relationship that is influenced by the researcher’s experience, her senses, and her embodied presence in the research context [44]. Thus, the first author went back and forth along the continuum of epistemic knowledge to gain a thick description and interpretation of knowledge [45]. This approach allowed for deep immersion and prolonged observation of the participants and spaces in each context to enhance an in-depth understanding of the phenomenon under study. Consequently, the researchers explored and pulled in significant relevant details that would otherwise be taken for granted. Reflective memos and discussions with the research team on various issues provided opportunities to challenge individual interpretations and mitigate potential bias.

### Research setting

The study was conducted in two different PHC facilities in rural and urban areas of two local government areas (LGAs) (Olamaboro and Dekina LGAs) of Kogi State, Nigeria, with populations ranging from 213,900 to 352,300 [46]. These PHCs are within five kilometres of residential areas, implying that the PHC facilities are available and accessible to most Nigerians [32]. These two PHCs were chosen because they offer comprehensive services ranging from antenatal, delivery, immunization, treatment of uncomplicated tropical diseases, and provision of antiretroviral medications.

### Participants

The study was conducted among Igala women of 18- 45 years of childbearing who were either pregnant and attended the antenatal clinic in a PHC facility in Kogi state or had given birth in a PHC facility in Kogi state within the last 12 months. All other ethnicities were excluded from the study, given that focused ethnographic research focuses on a population's subculture. The research was open to women of all sexual orientations and women who were single, divorced, separated and married. However, all study participants were married and had living partners. The purposive and snowballing techniques were used in different ways to recruit participants. First, through purposive sampling, the first author gained entrance to the antenatal and immunization health talks, with the assistance of the gatekeepers in each facility, where she invited women to participate in the study. Following the health talks, interested women met privately with the researcher, who explained the aim and objective of the study. Women who showed interest in the study were recruited after they obtained oral consent from their husbands to participate in the study. Through the snowballing technique, recruited women also referred other women with similar characteristics who met the inclusion criteria. See Table 1 for the demographic characteristics of women included in the study.

**Table 1:** Participants demographic characteristics

	**Number**	**Age**	**Marital status (Married, Single, Separated, divorced)**	**Parity**	**Educational qualifications**	**Religion**	**Occupation**
Rural Area Participant Observation (RA/PO),	*n* = 5	20–33	Married	1–4	High school diploma (5)	Christians (2) Muslims (3)	Housewives (5)
Urban Area Participant Observation (UA/PO),	*n* = 4	24–27	Married	1–4	Nil education (2) High school diploma (2)	Muslim (3) Christians (1)	Housewives (4)
Rural Area Interviews (RA/IDI),	*n* = 11	20–43	Married	2–6	Nil education (1) High school diploma (5) Associate degree (4) Bachelor’s degree (1)	Muslim (6) Christians (5)	Housewives (8) Business (1) Teaching (1) Nursing (1)
Urban Area Interviews (UA/IDI),	*n* = 10	19–40	Married	1–5	High school diploma (5) Associate degree (4) Bachelor’s degree (1)	Muslim (6) Christians (4)	Housewives (8) Public servant (1) Teaching (1)
Rural Area Focus Group discussions (RA/FGD),	*n* = 6	23–42	Married	1–4	High school diploma (2) Associate degree (3) Bachelor’s degree (1)	Muslim (3) Christians (3)	Business (3) Teaching (3)
Urban Area Focus Group discussions (UA/FGD)	*n* = 7	18–44	Married	1–5	High school diploma (4) Associate degree (2) Bachelor’s degree (1)	Muslim (6) Christians (1)	Business (3) Teaching (1) Public servant (2) Farmer (1)

Definitions: *RA/PO* Rural Area Participant Observation, *UA/PO* Urban Area Participant Observation, *RA/IDI* Rural Area Interviews , *UA/IDI* Urban Area Interviews, *RA/FGD* Rural Area Focus Group Discussions, *UA/FGD* Urban Area Focus Group Discussions

### Data collection

Data collection was conducted between August 2023 and November 2023, congruent with ethnographic methodology, to understand people in their natural environment. We used semi-structured one-on-one interviews, participant observations, and focused group discussions in the study.

### Participant observations

The first author employed two types of observation, starting with passive observation, which allowed for an in-depth understanding of the context, processes and relationships existing in the population. Later, a selective approach of participant observation, “observer as participant,” was instituted, allowing the researcher to follow up on nine women in the last trimester of pregnancy and four of those women during delivery. Observation flyers were posted in strategic places in each facility, and observation guides ensured boundaries of observation. The selective observation aimed to understand how much culture is integrated into antenatal and post-natal health talk, quality of care provided, approach to communication, support provided for women during pregnancy and delivery, respect of cultural preferences and verbal and non-verbal cues, and eye contact.

The observations ended when the researcher reached saturation, and no new data was generated to answer the research question. Thus, saturation was reached when the researcher repeatedly began to observe the same themes and patterns previously observed. Each observation lasted 4-5 hours but was longer on the days the researcher was observing a woman in labour. The participant observation yielded a total of 189 hours of observation. The observations were recorded in a field note by the first author and were transferred into a Microsoft Word™ document that was transcribed within 12 hours after the study to maintain the accuracy of the event [47].

### Interviews and focused group discussions

Recruitment for participation in one-on-one interviews, focus group discussion and selective participant observation, ran concurrently. With the assistance of the gatekeepers in each facility, the researcher was invited each week to share information about the research and invite women to participate in the study during antenatal and immunization schedules. Though the gatekeepers enhanced ease of entry to each facility, they were unaware of the participants interviewed during the study as there were clearly documented and discussed boundaries on their role in the study, which was duly signed by the two gatekeepers. For example, each gatekeeper did not have access to data, and did not suggest participants to the researcher, or interview participants.

Interested women who were attending the facility for antenatal and immunization clinics and women who were referred by participants usually met with the researcher privately in an office that was specifically assigned to the researcher. The first meeting with each participant focused on the researcher introducing herself and the research. The researcher started by describing the aim, purpose, objectives, potential benefits and harm related to the research, the participant's right to withdraw from the study, and audio recording of conversations with the participant’s permission. Issues of confidentiality and anonymity, as well as how the generated information from participants would be used, were also discussed with each participant. Thus, the researcher assured participants that their names, demographic characteristics and other sensitive information provided were protected and separated from study documents [48, 49]. Additionally, participants were informed that their names would be replaced with serial numbers, which will ensure that participants were not identified in the presentation of results and data analysis. Participants were also informed that their raw data would only be accessible to the study researchers.

During the conversation, participants asked diverse questions about the research and their participation, which were duly answered. Prospective participants who showed interest in the research were then informed to consult with their husbands at home, and if their husbands provided oral consent, they could sign the consent form and participate in the study. After obtaining consent from their husbands, women still interested in participating in the study were allowed to sign the consent form. Participants were scheduled for one-on-one interviews, focus group discussions and participant observation based on their availability and interest.

Interviews were conducted with 11 women in rural areas and ten women in urban areas who had recently given birth. Most of the interviews were conducted in a secure hall within both health facilities, ensuring privacy and comfort for the participants. Before commencing each interview, the researcher obtained participants' demographic characteristics using a questionnaire. The interview started with the researcher introducing herself and providing participants with information on the aim and purpose of the research. Oral consent and permission to record the interview were also obtained. Interviews were guided by an interview guide, informed by past literature reviews and themes generated during observations and validated by two authors who have lived among the Igalas for over 25 years. The researcher started the interview by asking preliminary questions such as “how has your day been today? The purpose was to establish rapport with participants and reduce anxiety associated with one-on-one interviews. The researcher continued with open ended semi-structured question, such as “What MHS are provided for you in this facility? Why do you access health services in this facility? How do the services here limit or satisfy your cultural and traditional expectations? How do health workers support and accommodate your cultural needs and preferences during pregnancy and delivery? How does the cost of maternal health services influence your use of facility care? How do cultural issues in this community influence your access to facility care? How do the equipment and amenities provided in this facility influence your use of this facility around pregnancy and childbirth? What roles do the community rulers play in ensuring women access MHS in this facility? What could be the challenges you have accessing MHS in this facility? What could the health workers and government do better to ensure your culture and traditions are respected throughout pregnancy and delivery? The researcher used probes to facilitate clarifications, elaborations, and a deeper explanation of previously provided responses [50]. Interviews lasted 30-50 minutes and were audio recorded, ensuring a thorough documentation of the discussions.

To enhance triangulation in the study, focus group discussions were held in the rural PHC facility with six women and seven women in the urban area in a mini hall within the PHC facilities. Given that women scheduled for focus group discussions were those accessing the facility for antenatal care and child immunization, the focus group discussions were held after the immunization of babies. The researcher collected each participant's demographic characteristics privately before ushering each participant into the hall for the discussion. The questions in the focus interview guide were a combination of the questions asked in one-on-one interviews and significant themes generated from observations and interviews, such as How does out-of-pocket payment for health services influence where you access care during pregnancy and childbirth? How does the quality of services provided here encourage women’s use of MHS? What role(s) do the elders in the community play in women’s use of facility care? What do you think the government and elders could do better to enhance your use of facility care? Focus group discussions lasted an average of one hour and fifteen minutes.

Interviews and focus group discussions were conducted mainly in English and a few in pidgin English (a simplified language of communication derived from the English language), which was spoken generally by the population and by the first and second authors. A back translation of all interview guides, consent, and recruitment documents was conducted [51] by the first author and checked by the second author, who both understand and speak English and pidgin English. All interviews and focus group discussions were conducted privately in a mini hall designated for the study, away from the activities of the facilities, with only the participants and the first author, which enhanced confidentiality and participants’ ability to freely and deeply share their experiences with the researcher [52]. In both interviews and focus group discussions, oral consent was obtained from participants to audio record the interviews and focus group discussion. 

### Data analysis

Data analysis began with data gathering and continued iteratively until the researcher gained a rich understanding and interpretation of the findings [41, 42]. There was a verbatim transcription of participant observations, field notes, reflective memo, and documents made freely available in both facilities, and all interviews and focus group discussions conducted in English. Back translation of a few interviews conducted in pidgin English was done and integrated into the analysis, which enriched the data. Data analysis followed the five steps described by Roper and Shapira [41], namely, (a) coding for descriptive labels, (b) sorting for patterns, (c) identification of outliers or negative cases, (d) generalizing with constructs and theories, and (e) memoing and reflective remarks. However, though these steps exist, data analysis in this study was not chronological as the researchers kept moving back and forth between the steps until a thick description and interpretation of the phenomenon under study was achieved [41, 42]. In the first step of coding for descriptive labels, the authors read the initial transcripts line by line to identify the codes running through each transcript. During this stage, the researchers met regularly to incorporate new themes in observations and early interviews in later interviews to enhance a rich interpretation of the data.

In the second stage of sorting of patterns, the authors began to identify the subthemes running through the codes, and their connections to the main themes identified. In the third stage of identifying outliers or negative cases, the researchers looked out for themes unrelated to the research question. These identified outliers were not discarded but were preserved to identify the relationship such outliers could have on the study. In the fourth stage of generalizing with constructs and theories, we compared our findings with existing literature on the phenomenon under study. The last stage of memoing and reflective remarks ensured our continuous reflexivity, which was enhanced by the reflective notes documented throughout the research process to enhance transparency and limit bias. Data saturation in this study was not instantaneous [42]. The first author gained insights from initial observations and interviews to expand future interviews and observations throughout the data-gathering process. Data saturation was reached when generated themes were supported with substantial data with no emerging new or contradictory information. See Table 2 for themes, codes, and excerpts of the study findings.

**Table 2:** Themes, codes and excerpts

Main themes	Subthemes	Excerpts
**Attitude of Healthcare Worker**	Supportive, kind, accommodating, respectful	the nurses they are very kind and they talk to us in a peaceful manner that will make you to come next time. Whenever you come here, you go home… happy. (RA/FGD/05)
	Birth companions during labour	They will have to drive him (husband) out and nurses will tell him that a man cannot enter the labor room, that he should wait outside (UA/FGD/05)
	Culturally appropriate care	You know, even when we come in labour and we bring our “Rubutu” (an Islamic concoction usually believed to promote speedy and safe delivery), we don’t need to hide it like in some other places (Urban Area/Observation/01)
	Abusive, slapping, unnecessary restraints, verbal abuse	For me,during my birth, there was a nurse that beat me and asked me if I wanted to kill my baby (UA/IDI/02)
**High Cost of Services**	High Cost of Services Free maternal health services	The government can also help by making registration for antenatal to be free (UA/FGD/01) Some are not privileged enough to take care of themselves, and so they feel that since I can get herbs that can last me for a week for a hundred naira, why will I go to the hospital that will charge me one thousand naira for ante-natal drugs? (UA/IDI/05)
**Issues Within PHC Facilities**	Limited Access to Far Away PHC Facilities	The government should provide places that are near… medical facilities for us … to walk across there when you are in labour (RA/IDI/01)
	PHC does Not Operate 24 Hours	Some of our clinics do not operate in the night, so when a woman is in labour, her first point of call will be, my neighbour is there…at least she has some knowledge (RA/IDI/01)
	Lack of Awareness of Available MHS in PHC Facilities	I don’t even know that they do delivery in this health centre (RA/Observation/02)
	Presence of Male skilled Attendants	I will not allow a male nurse to see my nakedness as that is our culture. (RA/IDI/O2) while you are pregnant…the religion only permit the male nurses to attend to you if you are in that situation… (RA/IDI/04) I don’t care if you are a man or a woman (UA/IDI/05)
	Poor Facility Infrastructure	This health centre does not have running water or light, and sometimes they cannot even do test for blood here (RA/Observation/ 04)
	Unavailability of Modern Equipment	We don’t have very good hospitals here that are well equipped with the necessary supplies that are needed to save lives so that people will not be transferred during pregnancy, labour and during emergency (RA/IDI/06)
	Capacity of Providers (Knowledge and Skills	My own reason is that sometimes you might come you might …meet a quack. I don’t think, anyone will like to leave his or her life to a quack nurse (RA/IDI/04)
**Contextual Cultural Issues in Communities**	Intergenerational Cultural Practices	Our tradition is important to us. My mother sat me down when I got pregnant and talked to me on several traditional things I have to respect and obey so that my pregnancy and delivery will be without problem (RA/Obseration/02)
	The Attitude of Elders in Communities	They don’t accept it because they base their decision on tradition, they say tradition is tradition… (RA/IDI/03)
	Lack of Community Engagement	…they will call our community, the elders, the husbands together, before taking the meeting and they will educate them, then advise them, for them to understand what they are talking about (RA/IDI/02)

### Trustworthiness

The study followed the criteria of credibility, transferability, dependability, and confirmability described by Lincoln and Guba [53]. We met the credibility criterion by piloting the research instruments with three women with the same characteristics as the participants to ensure the tools were valid and appropriate to the research question. Data from the pilot study were not included in the main data. The first author had a prolonged engagement in the context and deep immersion in the data to promote understanding and interpretation. The authors met continuously to debrief on the generated findings. Member checking was also done by the first author, who collected the data to reduce errors and ensure that data represents the participants' views. To meet the criterion of transferability, purposive and snowballing sampling techniques were employed to ensure participants were selected based on their experience with the phenomena of interest, which enriched the data. We also provided a detailed description of the data-gathering approach and ensured that research team members conducted data coding to meet the criterion of dependability. Several data triangulation approaches were employed to ensure confirmability, such as a detailed audit trail, reflective and field notes, participant observations, and focus group discussions, to enhance the confirmability of research findings and ensure a rich study.

## Results

**Fig. 1:**
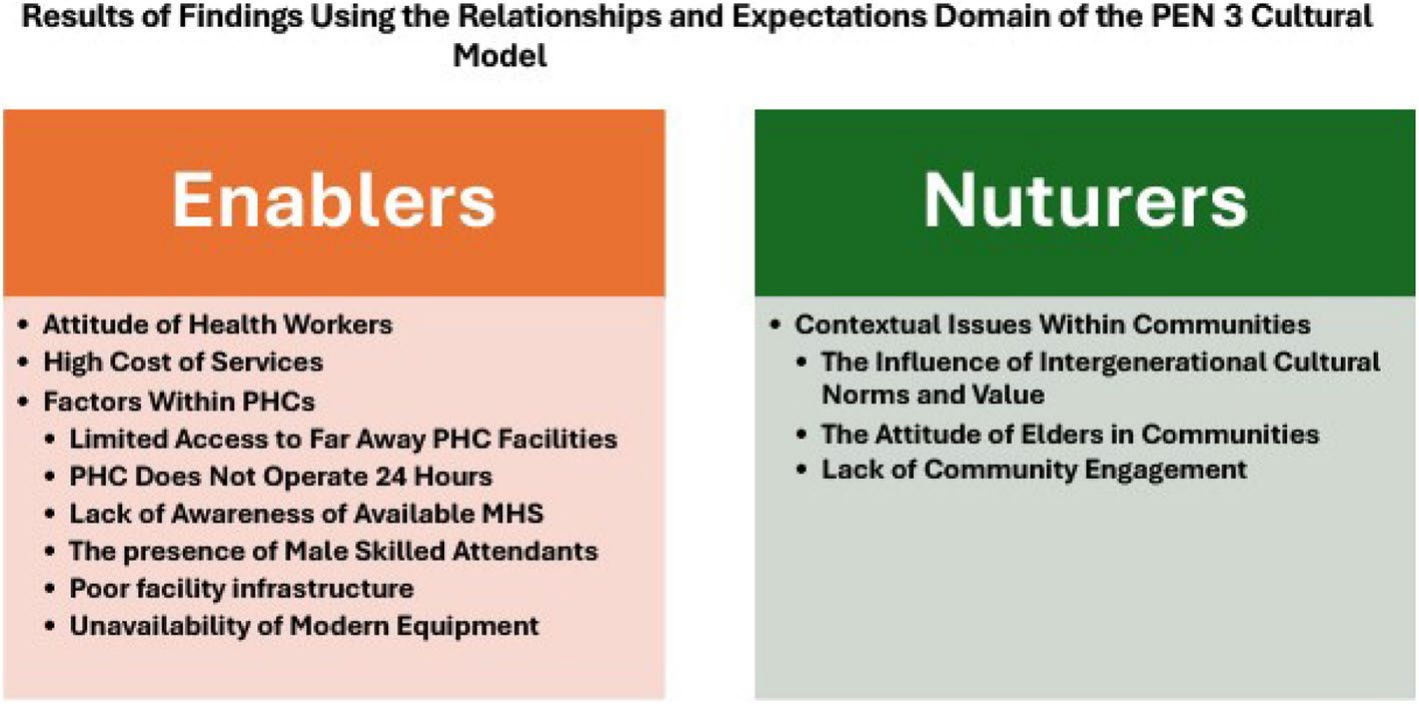
Results of findings using the relationship and expectations domain of the PEN 3 cultural model

Forty-three pregnant and nursing women of diverse demographic characteristics in education, religion, and location aged 18 to 44 years, with parity ranging from one to six, participated in the study. Using the construct of enablers and nurturers of the relationships and expectations domain of the PEN-3 cultural model, four themes were generated: The attitude of health workers, The high cost of services, Factors within PHC facilities and Contextual issues within communities. The theme, Attitude of healthcare workers and The presence of male skilled attendants, a subtheme in the theme of Factors within PHC, were found to be both positive and negative enablers that either facilitated or limited women's engagement in cultural practices and use of facility care. However, other themes, such as the High cost of facility care, subthemes in the theme of Factors within PHC facilities, such as Limited access to far away PHC facilities, PHC does not operate 24 Hours, Lack of awareness of available MHS, Poor facility infrastructure, Unavailability of modern equipment and subthemes in the theme of Contextual issues within communities such as The influence of intergenerational cultural norms and value, The attitude of elders in communities and Lack of community engagement were found to be negative enablers and nurturers that limit women’s access to facility care and enhanced engagement in harmful cultural practices. See Fig. 1 for themes and subthemes generated using the PEN 3 cultural model.

### The relationship and expectation domain of the PEN 3 cultural model

Using the relationship and expectation domain, we explored the societal or structural resources, such as the health system, socioeconomic factors, and contextual issues, that facilitate or limit women’s access to MHS in PHC facilities.

#### Enablers

Under this construct, we found that themes such as The attitude of health workers and The presence of male skilled attendants, a subtheme generated under Factors within PHC facilities, were significant positive and negative enablers of cultural norms and practices that either limited or facilitated the use of MHS in PHC facilities. However, the theme, High Cost of Services, and subthemes under Factors within PHC facilities such as Limited access to far away PHC facilities, PHC does not operate 24 hours, Lack of awareness of available MHS, Poor facility infrastructure and Unavailability of modern equipment were negative enablers that limited women’s access to PHC facilities and engagement in harmful cultural norms and practices.

#### The attitude of health workers

We found that the attitude of healthcare workers was both a positive and a negative enabler that facilitated and limited women’s use of MHS in PHC facilities. As a positive enabler, women narrated that the care providers' friendly, kind, accommodating, and respectful attitude were the reasons for the continued use of MHS in PHC facilities. Many narrate that most health workers in the PHC facilities do not shout at them, scold or maltreat them, unlike in other health facilities. Such attitudes also extend to the labour period, where women receive significant support from health workers during labour and delivery, which they believe makes the pain of childbirth bearable.

… the nurses they are very kind and they talk to us in a peaceful manner that will make you to come next time. So, there is no day that you come here for immunization or antenatal care that you will go home with a heavy heart or as in you are hurt and angry. Whenever you come here, you go home… happy. (RA/FGD/05).

… the support they give us in the health facility, like this (mentions a PHC facility) that we are going to, …..what makes us to go there always is whenever we are doing labour, their nurse, they will be petting you, they will be holding you, they will be walking around petting you, that makes us to be going there always (RA/IDI/07).

A few women also narrated that they were allowed birth companions, such as their husbands and families, who support and encourage them during delivery. Women narrated that such support was significant as it encouraged bonding between women and their husbands, made the labour pain bearable, and increased the respect their husbands had for them when they watched them go through labour and give birth. However, most of the participants recounted that their husbands and families were not allowed into the labour room to support them emotionally and physically during labour.

They will have to drive him (husband) out and nurses will tell him that a man cannot enter the labor room, that he should wait outside and your wife will be fine. He said he knows, but he just wants one minute to pray with his wife (UA/FGD/05)

Women also recounted that, in most cases, health workers respect their cultural values and beliefs in care, which is important to them and enhances their continual use of such PHC facilities.

You know, even when we come in labour and we bring our “Rubutu” (an Islamic concoction usually believed to promote speedy and safe delivery), we don’t need to hide it like in some other places where they do not allow us to use it, they even help us and allow us to drink it and help rub some on our belly and private part, so why will I not come again when I am in labour” (Urban Area/Observation/01)

Women stress that most health workers from other ethnicities provide appropriate culture-focused care, have learned the Igala language, and communicate with patients in Igala land. However, some health workers are still not conversant with contextual cultural values in most PHC facilities. Women emphasize that such health workers must understand their cultural beliefs, values, and practices and apply these in care provision to establish trust, acceptance, and women's continued use of MHS in PHC facilities.

So, when you are in the midst of people you have to monitor them to know the ways of their life so that you can live the ways of their life, with that you can have unity. Maybe your way of life is different from their way of life, there will be no unity because they will see you as a stranger, you understand? So, they must understand us (RA/IDI/11).

However, as a negative enabler that could limit women's access to MHS, a few women narrated issues of abuse during the second stage of labour, such as slapping, unnecessary restraints, verbal abuse, and denial of a companion at birth. Women recount that, in most cases, many health workers interpret such abuse as a strategy to prevent birth complications in the second stage of labour and assist mothers to focus during delivery.

…during my birth, there was a nurse that beat me and asked me if I wanted to kill my baby or do I want my baby to drink water, that I should balance well and give birth and, so she was beating me, and I was asking in my mind that why is this nurse beating me not knowing that she was helping me. So, when I delivered, she came to me and asked me not to be angry of what she did to me in the labor room that she wanted to help me, I then told her that I wasn’t angry, that I was even happy she helped because if she didn’t, then I wouldn’t have known what will happen to my baby (UA/IDI/02).

#### High cost of services

The high cost of MHS, which is unaffordable for most women, was found to be a negative enabler that limited women’s use of MHS in most PHC facilities. Moreover, most MHS are paid out of pocket, which is beyond the reach of many families and is responsible for women’s decision to seek cheaper options such as the use of herbal medicine and home deliveries. Many women do not use PHC facilities as they believe that the amount spent in facility care could be transferred to meet other basic pressing needs in the family, especially at this time in Nigeria, when feeding is a significant problem.

They feel health care is going to take more money from them. So, the little money they saved, they want to use it to buy baby things or to eat. Some are not privileged enough to take care of themselves, and so they feel that since I can get herbs that can last me for a week for a hundred naira, why will I go to the hospital that will charge me one thousand naira for ante-natal drugs? That is the main reason they do that here in Igala land (UA/IDI/05).

Sometimes you go to hospital…you have to pay…maybe high amount before you give birth. They will ask you to pay a lot of money, and some of them are not buoyant enough. That is why they chose to go for home delivery; sometimes you deliver at home if God helps you to deliver safely, they will say you should pay 5000 thousand naira, they prefer that one to the hospital because of the bill. If I see someone that attends to me at home, the price is less and she is perfect in what she is doing, I will go for that, irrespective of the help I got from the facility (RA/IDI/04).

However, according to some women, some health facilities provide the required MHS and allow women to make initial deposits and complete the payment at their convenience. Women narrate that such provision assists in reducing the burden of paying large sums of money at an instance that many may not have access to. Based on the economic situation in Nigeria, many women emphasize the need for free MHS to increase women’s use of facility care and enhance maternal health outcomes.

The government can also help by making registration for antenatal to be free. And also to reduce the money paid for delivery. That is the major thing to me (UA/FGD/01).

### Factors within PHC facilities

#### Limited access to far away PHC facilities

We also found that access to far-away PHC facilities was a negative enabler that limited the use of PHC and promoted the use of traditional birth attendants. While many PHC facilities are located within five kilometres of residential areas, women narrate that many traditional birth attendants live closer to them and are more accessible than most PHCs facilities. These traditional birth attendants provide culturally focused care and are cheaper to access than PHC facilities. Women stated the need for the government to build closer PHC facilities, providing essential services to women, especially in rural areas where most women have limited access to numerous PHC facilities. Women also stressed the need for the government to build accommodations for health workers in rural areas to enhance ease of access for women when labour starts at odd hours, which could reduce maternal and fetal mortality.

One of the reasons, my sister, that people access traditional birth attendant at times, is not even because of culture, it is because of nearness. The woman next door is a traditional birth attendant, so even if the thing happens in the night and I am feeling pain, I know I can rush to her… The government should provide places that are near… medical facilities for us … to walk across there when you are in labour (RA/IDI/01).

#### PHCs do not operate 24 hours

The lack of 24-hour services in some PHC facilities was seen as a negative enabler that limited access to facility care. Women narrated that some PHC facilities provide day shifts running for six to eight hours, which discourages women’s use of such facilities during labour. Based on this awareness, many women whose labour onset occurs at night, especially in rural areas, end up delivering with traditional birth attendants or with families who may have limited understanding or experience with conducting deliveries.

Some of our clinics do not operate at night, so when a woman is in labour, her first point of call will be, my neighbour is there…at least she has some knowledge (RA/IDI/01).

#### Lack of awareness of available MHS in PHC facilities

Lack of awareness of available MHS in PHC facilities was a significant negative enabler that limited access and use of services in many PHC facilities. Women emphasized the need for PHC facilities to raise awareness of the services they provide. Most women are unaware of the scope of services provided in most PHC facilities, which limits their use of such facilities.

I don’t even know that they do delivery in this health centre. I have been coming here for my antenatal and for immunization. It was when the health centre was near the market that I used to hear that people deliver there (RA/Observation/02).

#### Presence of skilled male attendants

The presence of male skilled attendants during labour was one of the themes generated during the research and was both a positive and a negative enabler. Women provided diverse views related to the presence of a male-skilled birth attendant during pregnancy and labour. For some, the presence of a male-skilled attendant is unacceptable as their culture does not permit another man to see their nakedness. Moreover, many women narrated that the presence of a male skilled attendant would make them shy and limit their ability to communicate freely during childbirth. In addition, women emphasize that male skilled attendants’ knowledge is limited to theoretical knowledge, lacking experiential knowledge when compared to female skilled birth attendants. Thus, women believe that male skilled attendants may not fully understand or adequately handle pregnancy and delivery. Many women narrated that the presence of a male skilled attendant is one of the reasons they would cease to access facility care and seek services with a traditional birth attendant or a facility with female skilled birth attendants.

I will not allow a male nurse to see my nakedness, as that is our culture. I don’t think I will open my body to a male health worker. If I must, I will not go back to that clinic again; rather, I will choose a place where a woman will take care of me (RA/IDI/O2).

However, many Muslim women narrated that while it is Islamically unacceptable for a male-skilled birth attendant to see the nakedness of a married woman, women during pregnancy and delivery are exempted due to their condition. As such, the Islamic religion does not frown at the presence of a male skilled attendant during pregnancy and birth.

while you are pregnant…the religion only permit the male nurses to attend to you if you are in that situation… because you are on a condition, so it permits that, but aside that, if another man should see your nakedness, it is abomination, traditionally and even Islamically (RA/IDI/04).

For many women, irrespective of religion, the gender of a skilled attendant is irrelevant. Women narrate that at the point of delivery, what really matters is having a safe delivery void of complications. Consequently, whoever attends to them is not an issue as long as the male skilled worker is trained, capable, and competent to handle labour and delivery.

My first child, it was a man that checked me …I didn’t know there was a difference between male and female. I thought they were all the same since it is his profession…I just want to be on the safer side… I don’t care if you are a man or a woman (UA/IDI/05).

#### Poor facility infrastructure

Women identified poor facility infrastructure as a negative enabler that limits access to PHC facilities. Women narrated that while staff in most PHCs facilities aim to provide quality care, several factors limit adequate and quality MHS provision in most PHC facilities. Many women also stressed that most health facilities, especially in rural areas, are run down without appropriate basic infrastructure and equipment to provide antenatal and delivery services.

This health centre does not have running water or light, and sometimes they cannot even test for blood here, so I am always afraid to come and deliver here. If my labour starts at night, where will they get light to see and work? So, I don’t come here (RA/Observation/ 04)

#### Unavailability of modern equipment

The unavailability of modern equipment in primary health facilities, especially in rural areas, was a negative enabler that limited women’s use of PHC facilities. Women narrated that the fear of losing their lives or their baby’s life hinders their use of the PHC facility in emergencies due to the lack of essential equipment and emergency services in most PHC facilities. In most cases, women are referred to bigger hospitals in the city to have a test or a procedure done for them, which raises questions about how such a facility could provide comprehensive services throughout pregnancy and delivery.

We don’t have very good hospitals here that are well equipped with the necessary supplies that are needed to save lives so that people will not be transferred during pregnancy, labour and during emergency (RA/IDI/06)..

#### Capacity of providers (knowledge and skills)

The lack of trained nurses and midwives in PHC facilities was another negative enabler limiting most women's facility care use. Most women recount that one of the reasons they do not access MHS in most PHC facilities is that most healthcare providers have limited training to care for women during pregnancy and to conduct deliveries. Many women narrate that while most facility care workers have some training, many health workers are not midwives who have been trained to attend to women during pregnancy and delivery. Thus, they lack the knowledge to understand emergencies and provide adequate care during emergencies. Consequently, due to such limitations, many women are afraid to access MHS in most PHC facilities.

My own reason is that sometimes you might come, you might …meet a quack. I don’t think, anyone will like to leave his or her life to a quack nurse or what ever to attend to. In a situation like that anybody will like to go to where he or she will be safe, sorry, where she will be safe and the baby will be safe (RA/IDI/04)

### Nurturers

#### Contextual issues within communities

We also found that within this construct, several contextual issues within communities, such as The Influence of intergenerational cultural norms and values, The attitude of elders in communities, and the Lack of community engagement, were found to be negative nurturers that facilitate cultural values and practices and limit women’s access to MHS in PHC facilities.

#### The influence of intergenerational cultural norms and values

Women’s beliefs and values of intergenerational cultural norms and practices are significant negative nuturers that limit access to facility care. Women narrated that such cultural norms are passed on from their forefathers and have guided them during pregnancy and delivery for generations. Such norms, they say, are important to them as they emphasize that even before Western civilization, several protective cultural practices and norms allowed them to experience pregnancy and deliveries without complication. Consequently, women narrated that, although they engage in Western medical practices, they do not intend to discard their inherited traditions, values, and norms that have been a way of life and have sustained and protected them all their lives.

Our tradition is important to us. My mother sat me down when I got pregnant and talked to me on several traditional things I have to respect and obey so that my pregnancy and delivery will be without problem. I know I can go to the hospital and obey all they tell me, but our traditional medicine is also good for me to take because oyibo (Western) medicine does not cure everything. Some women in our place here don’t even go to the hospital; they don’t value it, they visit old men and women in our place here to help them in this condition. There are some things our local medicine can cure that Oyibo medicine cannot cure. So if you decide to follow the oyibo, oyibo way and forget what we do here, you may lose your life and that of your baby (Observation/RA/03).

However, while many women narrated the significance of holding tenaciously to cultural values and practices, for some, such practices were not passed on to them and so are not known to them. Moreover, some believe that some traditional beliefs and practices, such as taking traditional medicines, could lead to complications that could lead to abortions, premature labour, and maternal and fetal death. Some women even narrated that though they may want to take traditional medicine, their initial reaction after taking such herbal concoction put them off trying native medicines. Consequently, based on their experience, they totally depend on medicines provided in PHC facilities.

#### The attitude of elders in communities

Many women in rural areas recounted the attitudes of elders in local communities who hold tenaciously and enforce traditional beliefs and practices that negatively influence maternal health outcomes. They indicated that these elders make laws on issues related to cultural norms and practices that must be respected in communities, such as traditional laws and values guiding the non-use of modern contraceptives. Women emphasized that such norms predispose women to unintended pregnancies, unsafe abortion, and pregnancy-related maternal mortality. Such norms and values have negative consequences if disobeyed and are believed to result in premature deaths in families. To make matters worse, these traditional norms and values are interwoven in religion as most elders in communities also hold high positions in churches or mosques.

Some of our elders, when any problem like that comes to them, some of them will be dodging …because they don’t want anything that will bring problem to their head. They don’t accept it because they base their decision on tradition, they say tradition is tradition… you cannot use your head to replace church matters because you will overlook your tradition (RA/IDI/03).

Women also stated that, in most cases, health workers are cautioned by leaders in communities to avoid stressing cultural issues that impinge on the use of MHS in PHC facilities, as such, could negatively affect the relationship with community leaders, their jobs, and their ability to operate effectively in such communities. Consequently, most health workers are not able to openly oppose dominant cultural or traditional issues that could impinge on the use of MHS in most communities where the study was conducted.

#### Lack of community engagement

The lack of community engagement was seen as a negative nurturer that negatively influence maternal health outcomes. Women emphasized the need for community engagement in communities that could bring an end to diverse cultural norms and practices that limit women's access to MHS and create awareness of the significance of MHS. Given the patriarchal setup of most communities in Nigeria, which is also reflected in the community where the study was conducted, women narrated the need for community engagement with community elders, religious leaders, and men who, in Igala ethnicity, are decision-makers. Women emphasize the need for the government and health workers to hold meetings with these elders and with women of childbearing age to emphasize the need for the use of MHS around childbirth to reduce maternal and fetal health complications.

They will call them together now, the government, they will call our community, the elders, the husbands together, before taking the meeting and they will educate them, then advise them, for them to understand what they are talking about (RA/IDI/02).

While most women agreed that community engagement with community elders could end cultural norms and practices that limit the use of MHS, some believe such engagements may have a limited effect on intergenerational cultural norms and values that are detrimental to maternal health. Some women believe that ending such traditional norms could attract the anger of the ‘gods’ or ancestors, which could be detrimental to the whole community.

## Discussion

This study was conducted to provide an in-depth understanding of facilitating and limiting factors of cultural norms and values influencing the use of MHS in PHC facilities among the Igalas in Kogi state, Nigeria. Using the enabler and nurturer constructs of the relationships and expectations domain of the PEN 3 cultural model allowed for a deep understanding of some of the factors that influence the use of MHS in Nigeria. One of the themes generated is the attitude of healthcare workers which, in most instances, showed that health workers were respectful, accommodating, and kind, in facilitating women’s use of facility care. Women further emphasized that accommodation of their traditional practices also encouraged them to access care in PHC facilities. Such respectful maternal care was also reported in a study in Ethiopia, where many of the women reported respectful care and assistance without physical and emotional abuse around childbirth [54], contrary to several studies that revealed significant abuse around childbirth in Ghana and South Africa [55, 56]. The reason for respectful maternal care in this study could be that most health workers are of Igala ethnicity, which enhances understanding of the language, values, and beliefs surrounding childbirth, promoting appropriate culture-centred and respectful care around childbirth.

A few of the women reported disrespectful treatment around childbirth, which is consistent with several studies that showed that the negative attitude of health workers is a significant deterrent to women’s use of MHS, especially in most SSA countries [55–57]. Abuse, such as slapping, unnecessary restraints, verbal abuse, and denial of a companion at birth, were described by women in the present study and were also revealed in studies conducted in Ghana, Kenya, and South Africa [55–58]. However, such actions were not seen as disrespectful by health workers but seen as protective acts [59, 60], as narrated in the present study, which is quite disturbing. Studies have also shown that such abuse is a significant determinant of home deliveries, the use of traditional birth attendants, and the avoidance of facility care [18, 58] and could potentially contribute to high maternal mortality [60, 61]. Though the [62] established recommendations to promote respective, supportive, and culturally sensitive care provision around childbirth, the effectiveness of such recommendations is limited in Nigeria due to limited policies guiding respectful maternal care, a weak health system, limited communications, and lack of a trusting relationship between care providers and the public [63]. Consequently, given the complex nature of respectful maternal care, a collaboration of stakeholders, policymakers, health system and care providers are needed to eliminate disrespectful maternal care [59, 63].

High cost of MHS was also a theme generated in the study that contributed to the non-use of facility care and home delivery as MHS is paid out of pocket (OOP) in most PHC facilities, which could be unaffordable for most low-income earners, especially in rural areas. Studies reveal that, since the launch of the Nigerian National Health Insurance Scheme (NHIS) in 2005, only 5% of the population has benefited from such insurance, as many states in Nigeria and public servants lack access to the scheme [64–66]. Thus, approximately 70% of the population's health care is financed through OOP expenditure [65, 67]. The NHIS provides comprehensive coverage of antenatal, delivery (vaginal and caesarian section), and post-natal services [66]; however, there remain hidden costs, such as medications not listed under NHIS, and cost of transportation not covered by the scheme. Studies in Central and Latin American countries and India are consistent with the findings in this study and have revealed that OOP for MHS has been found to decrease the use of facility care and increase maternal mortality, especially in socially marginalized communities [68, 69]. Though many women in this study narrated the need for free MHS to enhance women’s access to MHS, many studies emphasize the need to address multiple barriers that limit MHS access to women, including dominant contextual and cultural issues that limit MHS access, even when such services are made freely available [6, 70].The study also found issues of accessibility and limitation of human and material resources consistent with most PHC facilities in Nigeria, which was also revealed in several studies [19, 31, 71]. In Nigeria, a standard PHC staffing should consist of a physician, four nurse/midwives, a pharmacy technician, one medical record officer, a laboratory technician, a community health extension worker (CHEW), and six junior CHEWS [32, 72]. However, while this list may reflect the standard expectation for a functional PHC, studies reveal a shortage of nurses, midwives and doctors in most PHCs in Nigeria, with most PHC facilities manned by CHEWS, providing care beyond their jurisdiction and capacity even with limited training to manage pregnancy and delivery when compared to nurses and midwives [73–75]. Consequently, many women are predisposed to maternal health complications or preventable deaths due to the substandard MHS received in most PHC facilities [74]. Moreover, most PHC facilities are unable to provide comprehensive care due to inadequate infrastructure, limited material and medical resources, geographical barriers and limited funding of the health system in Nigeria [19, 31, 71]. Budgetary allocation to health in Nigeria is about the lowest in most SSA countries, hardly exceeding 7% of the total national budget, which is below the 2001 Abuja declaration of assigning at least 15% of the national budget to health [64, 76]. Thus, the meagre funding allocated to curative care leaves the PHC system largely underfunded to undertake a multidisciplinary and comprehensive maternal healthcare provision [64]. In addition, community engagement, designed to be integrated as part of PHC, is non-existent in most facilities, limiting communities’ participation in healthcare projects and programs to promote sustainable outcomes [31, 34]. Such limitations could account for why women prefer accessing traditional birth attendants due to ease of access, provision of culture-appropriate care and provision of cost-effective care when compared to PHC facilities [18, 21], as also seen in this present study. Consequently, the reinvigoration and financial strengthening of PHC facilities in Nigeria is critical to meet the rising population's demands through equitable and affordable services and availability of human and material resources, especially in rural areas where PHC facilities are the only available facility within reach.

We also found conflicting views about the presence of male skilled attendants in delivery. While many women narrated that such was against their culture and could limit their access to facility care, others were not bothered about the gender of the skilled attendant as far as such male skilled attendant had sufficient skills to manage obstetric issues and delivery. Our findings align with a study conducted in Kenya [77], where women were not bothered about the gender of care providers during labour as long as such skilled attendants had the necessary qualifications. Similar to our finding, studies in Ethiopia [78, 79] showed that many women were uncomfortable with a male skilled attendant at delivery. Given the conflicting findings, understanding women’s gender preferences in care provision is crucial to enhancing women’s access to MHS.

Similar to other studies in Ethiopia and Tanzania [78–80], contextual cultural norms passed on from generation to generation, such as the use of herbal concoctions, access to traditional birth attendants, and patriarchal and gender issues, were found to be significant factors influencing women’s use of MHS. However, several studies emphasized the need for community engagement, as was suggested by participants in this study, which could enhance women’s use of MHS and reduce maternal mortality. According to [81], community engagement enables individuals, groups, or organizations within a social context to participate and make decisions in the planning, designing, managing, and delivering of health interventions. Such engagement could allow stakeholders to understand a community’s priorities and ensure contextually appropriate health strategies tailored to the community’s cultural needs, given that cultural norms and social structures within communities, impact health behaviours and outcomes [82, 83]. Community engagement in Mozambique, Pakistan and India has enhanced the engagement of families, partners, communities and families in decision-making to support MHS tailored to the needs of the population, promoting increased understanding of pregnancy and birth complications, increased use of MHS and enhancing maternal health outcomes [84–86]. The Nigerian government could harness such strategies to ensure appropriate culturally focused MHS, which is adapted to the cultural expectations of the population.

## Strength and limitations

A strength of this study is the participation of women with diverse demographic characteristics in education, religion, age, parity, location, and occupation, which promoted a thick description and interpretation of the phenomenon of interest. Additionally, focused ethnographic research offered us diverse methods that enhanced triangulation and the study’s rigor. The PEN-cultural model was also beneficial, as it's use as a framework allowed us to capture significant facilitating and limiting factors of cultural values and norms that influence women’s use of MHS in PHC facilities. However, the study was conducted in PHC facilities alone, necessitating future exploration of women’s perspectives in secondary and tertiary health systems. Thus, further research could be employed to understand the perspectives of women who access secondary and tertiary health facilities in Nigeria.

## Policy statements

Though cultural beliefs and practices are significant factors that influence women’s use of MHS, not much is known about significant factors that facilitate or hinder these cultural norms and values regarding women’s use of MHS in PHC facilities. This study has brought to light numerous significant factors that could facilitate or hinder cultural norms and values with regard to the use of MHS in PHC facilities. Findings reveal that the attitude of health workers and culture-appropriate care are facilitating factors that influence women’s use of MHS, though few women reported abuse, which was insignificant to most health workers. Consequently, modules of respectful maternal care and culture-appropriate care could be introduced among health workers in PHC facilities, which is lacking in Nigeria to ensure respect, dignity, and valuing of women’s cultural preference in care provision, which could enhance women’s satisfaction, increased use of maternal health services and promote maternal health outcomes.

Additionally, the OOP payment for MHS is a significant factor that limits the use of services in PHC facilities and increases engagement in harmful traditional practices. Consequently, policies could look towards free MHS by making the NHIS available to individuals in rural areas to reduce maternal mortality. The state of PHC facilities in Nigeria is deplorable, given the lack of human and material resources needed for the provision of timely, adequate MHS due to limited health financing [19, 31, 71]. Consequently, stakeholders, policy makers, and the Nigerian government need to critically explore multiple approaches to ensure comprehensive MHS provision in PHC facilities. Additionally, community engagement is an important strategy that could enhance understanding of contextual and cultural issues, which could be harnessed to promote the provision of MHS that is responsive to communities' cultural needs [71–73]. Such community engagement, which has been effective in Asian and other African countries [87–89], could be harnessed to enhance maternal health outcomes in Nigeria.

## Conclusion

In this study we have provided a deep understanding of some of the facilitating and limiting factors of cultural values and norms that influence the use of MHS in PHC facilities. The use of the enabler and nurturer constructs of the relationships and expectations domain of the PEN 3 cultural model was significant as a framework for organizing our findings and understanding the facilitating and limiting factors of cultural norms and values. Using the PEN 3 cultural model, we found that the theme, attitude of healthcare workers was both a positive and a negative enabler that either facilitated or limited women’s use of MHS in PHC facilities. We also found that the high cost of MHS was a negative enabler that influenced women’s engagement in harmful cultural practices and nonuse of facility care.

Additionally, a subtheme of the factors within the health system, such as the presence of male skilled attendants, was both a positive and negative enabler that facilitated and limited women’s access to PHC facilities. However, other subthemes in the theme of factors within the health system, such as the lack of nearby PHCs, the lack of appropriate human and material resources, and lack of awareness of services provided in PHC facilities, are significant negative enablers that promote alternative cultural care-seeking behaviours and limited use of MHS in PHC facilities. Finally, contextual cultural issues, such as the influence of intergenerational cultural issues, the attitude of community elders, and the lack of community engagement, were dominant cultural nurturers that deepened cultural norms and limited the use of MHS in PHC facilities. Further studies are needed on approaches to enhance sustainable strategies that limit harmful cultural norms and practices and enhance MHS use. Additionally, the reinvigoration of PHC is critical through increased health financing, attention to material and human resources, and community engagement to promote comprehensive care delivery in communities.

### Supplementary Information

Supplementary Material 1.

## Data Availability

Data are available on the reasonable request from the corresponding author.

## Abbreviations

CHEWS: Community Health Extension Worker
FE: Focused Ethnography
IMNCH: Integrated Maternal, Newborn and Child Health Strategies
MHS: Maternal Health Services
MMR: Maternal Mortality Ratio
MSS: Midwives Service Scheme
NHIS: Nigerian National Health Insurance Scheme
OOP: Out-of-Pocket
PHC: Primary Health Care
SDG: Sustainable Development Goals
SBAs: Skilled Birth Attendants
SSA: Sub-Saharan Africa
UN: United Nations
UNICEF: United Nations Emergency Fund
WHO: World Health Organization
