# Supplementary material for: Facilitating and limiting factors of cultural norms influencing use of maternal health services in primary health care facilities in Kogi State, Nigeria; a focused ethnographic research on Igala women
Source: BMC Pregnancy Childbirth. 2024 Aug 27;24:555. doi: 10.1186/s12884-024-06747-x (PMC11348738; doi:10.1186/s12884-024-06747-x)
Supplement: Supplementary file 1 — Supplementary Material 1. [file 12884_2024_6747_MOESM1_ESM.docx]

**Interview Guide (One-on-One Interview)**

**Preambles**

Introduce yourself to the participant.

Describe the purpose of the interview and how information will be used.

Obtain oral consent.

Provide instructions guiding the interview.

Interview place:

Interview date:

Start time:

End time:

**Information Sheet**

| Participant Identification |  |
| --- | --- |
| Age |  |
| Number of pregnancies |  |
| Number of births |  |
| Number of children |  |
| Educational level |  |
| Occupation |  |
| Family income |  |
| Religion |  |
| Place of residence |  |

**General question:**

How has your day been today**?**

Can you tell me a little bit about yourself?

**Maternal Health Services**

**1.** Can you tell me your understanding of health services provided for women during

pregnancy and after delivery?

**2.** What are the services provided for you in this facility?

**Probes**

a. What ante-natal services are provided for you during pregnancy?

b. What services are provided for you during delivery?

c. Could you tell me the services provided for you from the time of delivery to six weeks after

delivery?

**Health personnel**

1. How do health workers support you during pregnancy?

2. What support do health workers provide you during and after delivery?

3. What understanding do health workers have of the cultural norms in this community?

4. How do health workers accommodate your traditional beliefs and practices during pregnancy?

5. How do health workers accommodate your traditional beliefs and practices when you are in labour?

6. How supportive are nurses in encouraging or discouraging these beliefs and practices?

7. How does health workers' support affect your use of maternal health services?

8. How do health workers' attitudes encourage you to continue using this facility during pregnancy and birth?

9. What could discourage you from using this facility during pregnancy and childbirth

**Cost of Maternal Health Services**

1. Which aspect of maternal health services do you pay for?

**Probes**: a. antenatal, b. laboratory tests, c. scanning, d. doctor’s consultation, e., delivery, f. postnatal services?

2. How do you pay for maternal health services provided for you in this facility?

**Probes: a.** Out-of-pocket payment, **b.** social insurance, **c.** free?

3. How does the cost of maternal health services influence your use of facility care during pregnancy?

4. How does the cost of maternal health services in this facility influence your use of this facility for delivery and postnatal care?

5. How does the cost of facility care encourage you to seek alternative health care services

Probes: Traditional birth attendants? b. Herbalist?

**Health System Factors**

1. How close is this health facility to your place of residence

**Probes a.** Within walking distance, **b.** within driving distance

2. How do the features, items, and amenities in this facility encourage you to use this health facility during pregnancy and delivery?

**Probes** **a.** health workers, **b.** equipment, **c.** medication and supplies, **d.** location of the health facility, **e.,** services provided. **f.** physical structure, **g.** environment

3. 2. How do the features, items, and amenities in this facility discourage you from using this health facility during pregnancy and delivery?

**Probes** **a.** health workers, **b.** equipment, **c.** medication and supplies, **d.** location of the health facility, **e.,** services provided. **f.** physical structure, **g.** environment

**Contextual Cultural Issues**

1. How do cultural issues and factors in this community encourage your access to facility care?

2. How do cultural issues and factors in the community hinder your access to facility care?

**Probes a.** cultural norms and values**, b.** community leaders, **c.** religion

3. What role do community leaders play to ensure women access quality maternal health services in health facilities?

**Concluding questions**

1. What do you think health workers could do more to serve you better?

2. What could the health workers do better to respect your cultural values during pregnancy and childbirth?

3. What could the community elders and heads do better to enhance your use of maternal health services?

4. What role can the government play in ensuring that women in this community use the facility better during pregnancy and delivery?

5, What could the government do better to ensure your cultural values are respected during pregnancy and childbirth?

**Probes a.** in the community, **b.** in the health facility

6. Is there anything more that I have not said that you would want to tell me?

Appreciate the participant.

End the interview.

**Guide for Focus Group Discussions**

**Preambles**

Introduce yourself to the participants.

Describe the purpose of the focused group discussion and how information will be used.

Obtain oral consent.

Provide instructions congruent with focus group discussions.

Focus group discussion context:

Focus Group discussion date:

Start time:

End time:

**Information Sheet**

| Participant Identification |  |
| --- | --- |
| Age |  |
| Number of pregnancies |  |
| Number of births |  |
| Number of children |  |
| Educational level |  |
| Occupation |  |
| Family income |  |
| Religion |  |
| Place of residence |  |

**General question:**

How has your day been today**?**

**Maternal Health Services**

**1.** Can you tell me your understanding of health services provided for women during

pregnancy and after delivery?

**2.** What are the services provided for you in this facility?

**Probes**

a. What ante-natal services are provided for you during pregnancy?

b. What services are provided for you during delivery?

c. Could you tell me the services provided for you from the time of delivery to six weeks after

delivery?

**Health personnel**

1. How do health workers support you during pregnancy?

2. What support do health workers provide you during and after delivery?

3. What understanding do health workers have of the cultural norms in this community?

4. How do health workers accommodate your traditional beliefs and practices during pregnancy?

5. How do health workers accommodate your traditional beliefs and practices when you are in labour?

6. How supportive are nurses in encouraging or discouraging your traditional beliefs and practices?

7. **What could nurses do better to accommodate your traditional beliefs and practices during pregnancy?**

8**. What could nurses do better to accommodate your traditional beliefs and practices during and after delivery?**

9. How does health workers' support affect your use of MHS?

10. How do health workers' attitudes encourage you to continue using this facility during pregnancy and birth?

10. What could discourage you from using this facility during pregnancy and childbirth

**Socio-economic Factors**

**1.** Which aspect of maternal health services do you pay for?

**Probes**: a. antenatal, b. laboratory tests, c. scanning, d. doctor’s consultation, e., delivery, f. postnatal services?

2. How do you pay for maternal health services provided for you in this facility?

**Probes: a.** Out-of-pocket payment, **b.** social insurance, **c.** free?

3. How does the cost of maternal health services influence your use of facility care during pregnancy?

4. How does the cost of maternal health services in this facility influence your use of this facility for delivery and postnatal care?

5. How does the cost of facility care encourage you to seek alternative health care services

Probes: Traditional birth attendants? b. Herbalist?

**6. What role does the facility play in assisting you with the bills for maternal health services?**

**Issues Within Health Facilities**

1. How close is this health facility to your place of residence

2. How do the features, items, and amenities in this facility encourage you to use this health facility during pregnancy and delivery?

**Probes** **a.** health workers, **b.** equipment, **c.** medication and supplies, **d.** location of the health facility, **e.** services provided. **f.** physical structure, **g.** environment

3. 2. How do the features, items, and amenities in this facility discourage you from using this health facility during pregnancy and delivery?

**Probes** **a.** health workers, **b.** equipment, **c.** medication and supplies, **d.** location of the health facility, **e.** services provided. **f.** physical structure, **g.** environment

**Contextual Issues in the Community**

1. How do cultural issues and factors in this community encourage or hinder your access to facility care?

2. How do community leaders ensure women access quality maternal health services in health facilities?

**Concluding questions**

What do you think health workers could do more to serve you better?

2. What could the health workers do better to respect your cultural values during pregnancy and childbirth?

3. **How else do you think the health facilities could support your traditional beliefs and norms to ensure you access facility care?**

4. What could the community elders and heads do better to enhance your use of maternal health services?

5. What role can the government play in ensuring that women in this community use the facility better during pregnancy and delivery?

**Probes a.** in the community, **b.** in the health facility

6. What could the government do better to ensure your cultural values are respected during pregnancy and childbirth?

7. Is there anything more that I have not said that you would want to tell me?

Appreciate the participants.

End the focus group discussion.
